# The role of probiotics, prebiotics, and postbiotics: cellular and molecular pathways activated on glial cells in Alzheimer’s disease

**DOI:** 10.3389/fnins.2025.1598011

**Published:** 2025-06-25

**Authors:** Aleidy Patricio-Martínez, Felipe Patricio, Edgar Macuil-Chapuli, Enoc Álvaro Martínez-Juárez, Steven Flores-Díaz, María Lilia Cedillo-Ramírez, Ilhuicamina Daniel Limón

**Affiliations:** ^1^Laboratorio de Neurofarmacología-Facultad de Ciencias Químicas-Benemérita Universidad Autónoma de Puebla, Puebla, Mexico; ^2^Facultad de Ciencias Biológicas-Benemérita Universidad Autónoma de Puebla, Puebla, Mexico; ^3^Facultad de Medicina Veterinaria y Zootecnia, Benemérita Universidad Autónoma de Puebla, Tecamachalco, Puebla, Mexico; ^4^Centro de Detección Biomolecular, Benemérita Universidad Autónoma de Puebla, Puebla, Mexico

**Keywords:** prebiotic, probiotic, postbiotic, glial cells, neuroinflammation, Alzheimer’s disease

## Abstract

Supplementation with prebiotics and probiotics can modulate the intestinal microbiota, returning it to a more physiological state; therefore, they can be considered as a possible treatment in many prevalent conditions, including neurodegenerative diseases. Alzheimer’s disease (AD) is the most common form of dementia, accounting for 60 to 70% of cases. The neuropathological features of AD include neuritic plaques (extracellular deposits of the beta-amyloid protein, Aβ), neurofibrillary tangles (resulting from hyperphosphorylation of the tau protein), a predominantly cholinergic synaptic decrease, and the presence of inflammatory markers, all these characteristics together trigger the neurodegenerative process and cognitive deterioration. The etiology of AD is multifactorial, however, in recent years evidence has been shown on the significant association between dysbiosis, neuroinflammation, and neurodegeneration. In the present review, we will discuss the role of gut microbiota in the pathogenesis of AD, as well as the underlying mechanisms that trigger the use of probiotics, prebiotics, and postbiotics in neuroinflammation. Our attention will focus on the cellular and molecular mechanisms triggered by astrocytes and microglia, cells involved in mediating neuroinflammation and neurodegeneration in AD.

## 1 Introduction

Dementia affected an estimated 57.4 million individuals globally in 2019, a number projected to rise to 83.2 million by 2030, 116 million by 2040, and 152.8 million by 2050 (GBD 2019 Dementia Forecasting Collaborators, 2022). Dementia leads to progressive cognitive and functional decline over time and is a leading cause of disability and dependence worldwide. Alzheimer’s disease (AD) is the most common form of dementia, accounting for between 60 and 70% of global dementia cases (WHO, 2023).

In recent years, intestinal health has been studied, since it is significantly influenced by the microbiota, which is largely composed of microorganisms and resides mainly in the gastrointestinal tract. Several studies have linked intestinal microbiota’s role with AD’s pathogenesis since patients with AD and mild cognitive impairment show a lower index of intestinal microbiota diversity than healthy control patients (Aziz et al., 2024; Xi et al., 2021). Notably, consistent microbial alterations in both animal models and human patients with AD include reductions in *Firmicutes* and *Bifidobacterium*, alongside increased abundance of genera such as *Bacteroides*, *Akkermansia*, and *Lactobacillus*, suggesting a disease-associated dysbiotic signature (Borrego-Ruiz and Borrego, 2024; Kang et al., 2024). To provide a clearer overview of the gut microbiota profile associated with AD, Table 1 summarizes representative microbial taxa that are consistently altered in both experimental models and patients. These dysbiotic patterns support the hypothesis that specific microbiome shifts may contribute to or reflect neurodegenerative progression.

**Table 1:** Representative microbiota alterations reported in Alzheimer’s disease models and patients.

Microbial Taxa	Changes	Observed species	References
*Akkermansia*	↑/↓ Mixed findings	Humans and mice	Kaiyrlykyzy et al. (2022), Ou et al. (2020), Wang et al. (2025)
*Bacteroides*	↑/↓ Mixed findings	Humans and mice	Liu et al. (2025), Xia et al. (2023)
*Lactobacillus*	↑/↓ Mixed findings	Humans and mice	Liu et al. (2025), Zhang S. et al. (2023), Zhang W. et al. (2023)
*Firmicutes*	↓ Decreased	Humans and mice	Kozhakhmetov et al. (2024), Manfredi et al. (2025)
*Bifidobacterium*	↓ Decreased	Humans and mice	Kozhakhmetov et al. (2024), Ma N. et al. (2023), Ma X. et al. (2023)
*Ruminococcus*	↓ Decreased	Human patients	Yamashiro et al. (2024)
*Escherichia/Shigella*	↑ Increased	Human patients	Chen et al. (2023)

These observations underscore the role of the microbiota-gut-brain axis (MGBA), a complex communication network between the gastrointestinal system (GIS) and the central nervous system (CNS), integrating the autonomic nervous system (ANS), the enteric nervous system (Carabotti et al., 2015; Hulme et al., 2022), endocrine pathways (Cussotto et al., 2018; Wachsmuth et al., 2022), and immune responses (Fung, 2020; Powell et al., 2017).

Experimental studies using animal models have illuminated the role of the MGBA in neurodegeneration, including AD (Hung et al., 2022; Liu et al., 2024). Approaches such as germ-free animals, probiotic administration, antibiotic treatment, and infection models have demonstrated the microbiota’s modulatory effects on neurodegenerative pathways (Bravo et al., 2012). A disturbance of the MGBA is implicated in the pathophysiology of gastrointestinal (GI) disorders, such as irritable bowel syndrome (IBS), Crohn’s disease (CD), and ulcerative colitis (UC), which represent a group of disorders. Chronic and severely debilitating (Tang et al., 2021; Tavakoli et al., 2021). The vagus nerve (VN) is the main component of the parasympathetic nervous system. It can detect microbiota, transferring this intestinal information to the central nervous system where it is integrated and then generating an adapted or inappropriate response; which could perpetuate a pathological condition of the digestive tract or promote neurodegenerative disorders (Eisenstein, 2016; Tse, 2017).

Emerging evidence suggests that MGBA modulation could offer novel avenues for the early diagnosis and treatment of neurodegenerative diseases, including AD (Liu et al., 2023). This review explores the latest findings on the MGBA, emphasizing the roles of prebiotics, probiotics, and postbiotics in modulating neuronal and glial activity, and their molecular mechanisms in AD.

### 1.1 Overview of the microbiota-gut-brain axis

The gut microbiota plays a critical role in the maturation of the immune system, particularly during the early stages of human development (Pantazi et al., 2023). From birth, the infant immune system evolves alongside changes in the microbiota, which are shaped by dietary and environmental factors (Zhang H. et al., 2022). Maternal influences and environmental exposures during pregnancy and lactation significantly impact the development of the infant’s intestinal microbiota (Nyangahu and Jaspan, 2019). By the age of three, the microbiota typically stabilizes, adopting a composition similar to that of an adult (Derrien et al., 2019; Yatsunenko et al., 2012). However, alterations during these critical early stages may have long-term health implications, predisposing individuals to inflammatory, immunological, and neurological disorders (Maciel-Fiuza et al., 2023; Sittipo et al., 2022). These disruptions can persist into old age, exacerbated by stress and contributing to dysbiosis (Zhao M. et al., 2023).

Dysbiosis, defined as an imbalance between beneficial and pathogenic microorganisms within the gut microbiota (Petersen and Round, 2014), is closely linked to the MGBA and associated with neurodegenerative diseases such as Parkinson’s disease (PD) (Cersosimo and Benarroch, 2012; Scheperjans et al., 2015). A notable example of this connection is constipation, affecting up to 80% of PD patients and often preceding the onset of motor symptoms (Edwards et al., 1992; Klann et al., 2021). Similarly, individuals with IBS characterized by dysbiosis face an increased risk of developing dementia, including AD (Chen et al., 2016). Intriguingly, certain bacteria, such as *BaGBcillus subtilis* and *Escherichia coli*, produce beta-amyloid proteins, which are implicated in Alzheimer’s pathology (Asti and Gioglio, 2014; Hill and Lukiw, 2015). These proteins may penetrate the intestinal barrier, enter the bloodstream, and potentially contribute to neurodegenerative processes (Friedland and Chapman, 2017).

**Figure 1:**
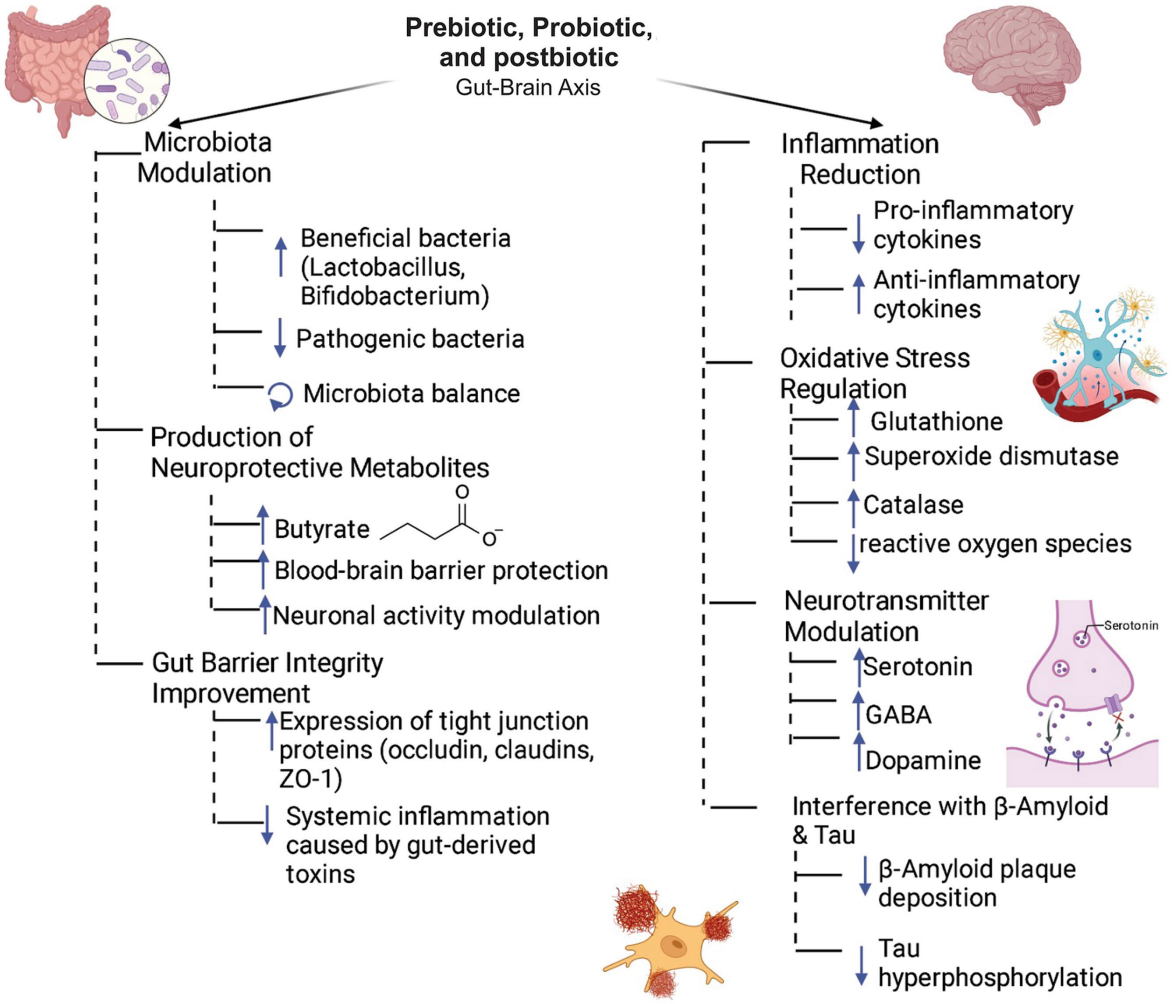
Role of prebiotics, probiotics, and postbiotics in the gut-brain axis. The synergistic interplay between prebiotics, probiotics, and postbiotics fosters intestinal microbiota homeostasis by selectively enhancing the growth of beneficial microbial taxa while suppressing pathogenic populations. This microbial modulation is closely linked to elevated production of neuroactive metabolites, notably butyrate, which has been shown to fortify the integrity of the blood–brain barrier (BBB) and modulate neuronal activity. In parallel, these bioactive compounds enhance intestinal barrier function by upregulating the expression of tight junction proteins, including occludins, claudins, and zonula occludens-1 (ZO-1). Systemically, they activate neuroprotective pathways by modulating immune responses, balancing pro- and anti-inflammatory cytokines, attenuating oxidative stress via upregulation of antioxidant enzymes and regulating the biosynthesis and release of key neurotransmitters such as serotonin, GABA, and dopamine. Collectively, these mechanisms contribute to the attenuation of neuropathological hallmarks associated with Alzheimer’s disease, including β-amyloid plaque accumulation and neurofibrillary tangle formation.

Maintaining a stable gut microbiota is essential for host protection against pathogens and for preserving homeostasis, including immune regulation (Wu and Wu, 2012). Disruptions in gut microbiota—induced by factors such as dietary changes, antibiotic use, aging, or infections—can lead to a spectrum of inflammatory, metabolic, and pathogenic conditions, including neurodegenerative diseases (Giau et al., 2018). These disturbances affect the MGBA through mechanisms involving the VN, the neuroendocrine system, and metabolites like short-chain fatty acids (SCFAs), which are carboxylic acids with aliphatic tails of 1 to 6 carbon atoms, which collectively influence mood, cognition, and overall neurological health (Ullah et al., 2023) (Figure 1).

Given the gut microbiota’s profound influence on multiple physiological systems, therapeutic interventions such as fecal microbiota transplants, probiotics, prebiotics, and postbiotics offer promising avenues for improving gut health and reducing the risk of neurological disorders. Further research is essential to understand their mechanisms and potential applications in clinical practice.

### 1.2 Characteristics of microbiota

The human microbiota consists of an estimated 10 to 100 trillion symbiotic microbial cells, primarily residing in the intestine, where bacteria predominate. In contrast, the term “microbiome” encompasses the collective genomes of these microbial communities (Ursell et al., 2012; Zhu et al., 2010). Global research efforts have sought to decipher the intricate roles these microorganisms play and their profound impact on human health.

The intestinal microbiota is essential for various physiological processes, including nutrient absorption, fermentation, host metabolism, and vitamin synthesis (de Vos et al., 2022). Additionally, it serves a protective role by competing with pathogens for resources and modulating the immune system (Belkaid and Hand, 2014). Beyond these core functions, the microbiota is a key regulator of oxidative and inflammatory processes and acts as a mediator in the bidirectional communication between the gut and the brain, forming the MGBA (Goyal et al., 2021; Shandilya et al., 2022).

Inflammation, the body’s response to harmful stimuli, is a critical mechanism for restoring homeostasis (Chen et al., 2018). However, chronic inflammation in various organs has been implicated in the progression of degenerative diseases (Baechle et al., 2023; Zhang W. et al., 2023). In particular, systemic inflammation involving the MGBA can lead to neuroinflammation, potentially linking the intestinal microbiota to neurodegenerative disorders.

Recent studies have highlighted the role of the microbiota in AD, emphasizing the importance of dietary intervations and the use of prebiotics, probiotics, and postbiotics (Peddinti et al., 2024; Seo and Holtzman, 2024). These approaches have demonstrated promise in modulating inflammation and enhancing the function of glial cells in the CNS.

Dietary interventions targeting the gut microbiota—including prebiotics, probiotics, and postbiotics—have demonstrated potential in modulating inflammation and enhancing glial cell function in the CNS.

#### 1.2.1 Prebiotics

In 2017, the International Scientific Association for Probiotics and Prebiotics (ISAPP) published a consensus statement defining a prebiotic as “a substrate that is selectively utilized by host microorganisms conferring a health benefit” (Gibson et al., 2017). This definition underscores the specific structure and composition of prebiotics, which is essential in the context of the microbiota’s complex functional ecosystems. Within these ecosystems, microorganisms metabolize carbohydrates, proteins, and certain fats, producing metabolites that positively influence host health (Peredo-Lovillo et al., 2020; Sanders et al., 2019).

While most prebiotics are carbohydrate-derived, other compounds such as polyphenols and polyunsaturated fatty acids also exhibit prebiotic activity (Peluso et al., 2014; Plamada and Vodnar, 2021). The structural characteristics of these compounds—such as monomer composition, bond type, and chain length—critically determine their bacterial degradation and utilization (Wang et al., 2020).

Carbohydrate-derived prebiotics, such as galactooligosaccharides (GOS), fructooligosaccharides (FOS), and inulin-type fructans (ITF), have been extensively studied for their microbial specificity (Davani-Davari et al., 2019). For instance, ITF from chicory has a simpler structure compared to the branched ITF from agave, resulting in differential degradation by microbial species (Mueller et al., 2016). Effective bacterial utilization of prebiotics requires specialized enzymes for degradation and transport mechanisms for internalization.

Additionally, (poly)phenols—a diverse class of compounds characterized by phenolic rings—offer unique prebiotic potential (Arfaoui, 2021). With over 10,000 types classified into subgroups such as tannins and flavonoids, their stability and biological activity depend on factors like pH and ionic strength (Oliveira Filho et al., 2021).

**Figure 2:**
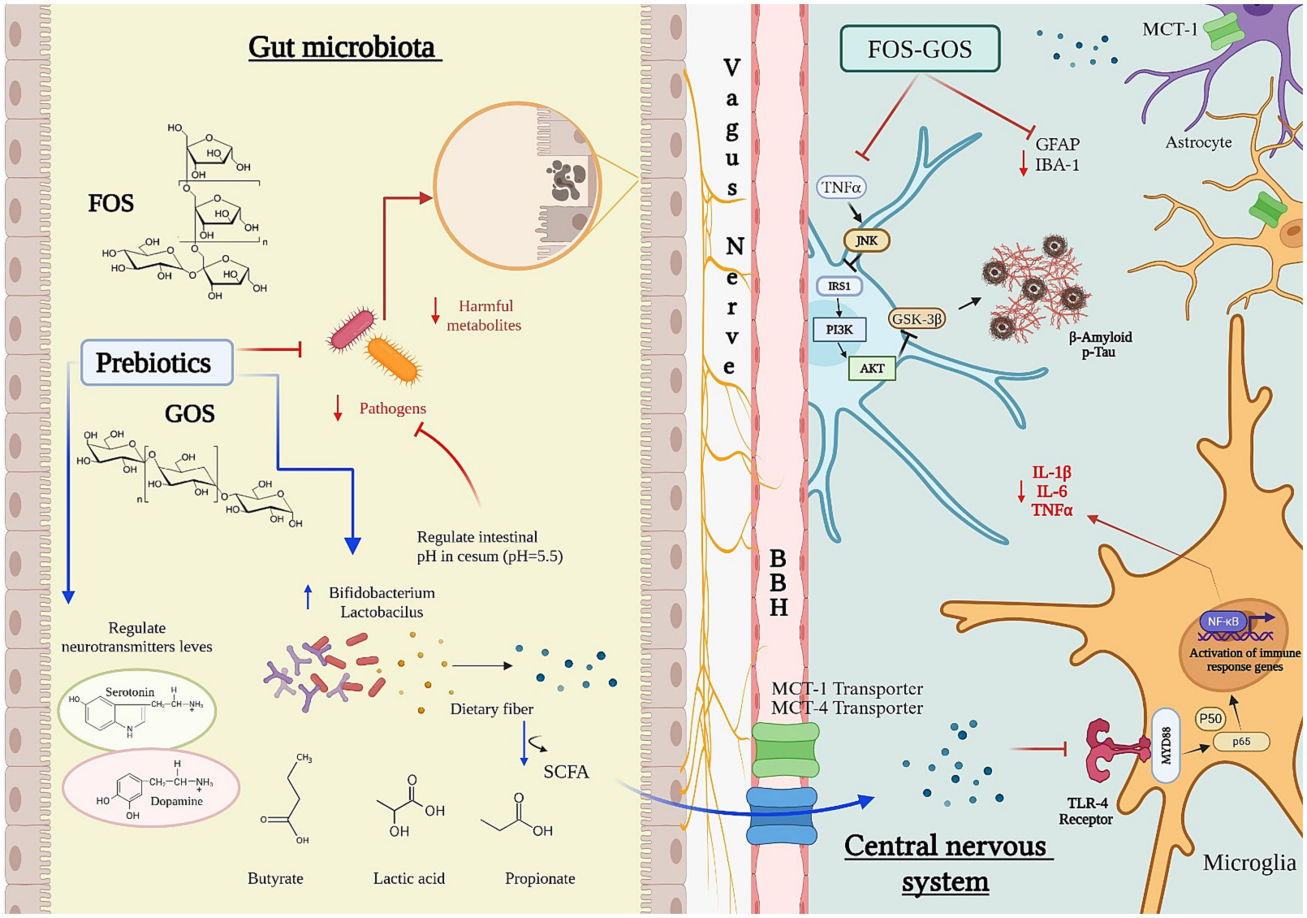
Influence of prebiotics on the microbiota–gut–brain axis. Fructooligosaccharides (FOS) and galactooligosaccharides (GOS) promote the growth of beneficial gut bacteria such as Bifidobacterium and Lactobacillus, enhancing microbial production of neurotransmitters including serotonin and dopamine. Fermentation of FOS and GOS by the gut microbiota and/or probiotics generates short-chain fatty acids (SCFAs)—notably butyrate, lactate, and propionate—that lower colonic pH and shape microbial composition, thereby influencing SCFA output. Most SCFAs are absorbed in the proximal colon via bicarbonate exchange, contributing to pH homeostasis. Cecal pH (~5.5) is consequently lower than rectal pH (~6.5), limiting the proliferation of acid-sensitive pathogens. SCFAs cross the blood–brain barrier (BBB) via monocarboxylate transporters (MCT1 and MCT4), with MCT1—expressed in cortical and hippocampal astrocytes—showing higher substrate specificity for acetate and butyrate. Through BBB- and vagus-mediated pathways, prebiotics modulate glial and microglial activity, suppressing inflammatory cytokines (IL-1β, IL-6, TNF-α) and reducing the accumulation of neurodegenerative markers such as amyloid-β and phosphorylated tau. SCFAs also trigger microglial immune gene expression via Toll-like receptor 4 (TLR4) and NF-κB signaling, contributing to neuroprotection and central immune regulation.

After ingestion, only a small fraction of (poly)phenols is absorbed in the upper GI tract. The majority reaches the large intestine, where it interacts with the microbiota and undergoes metabolism, releasing bioactive metabolites (Mosele et al., 2015). This interaction not only alters microbial composition but also provides health benefits, such as reducing inflammation and oxidative stress (Cardona et al., 2013; Plamada and Vodnar, 2021) (Figure 2).

#### 1.2.2 Probiotics

On October 23, 2013, the ISAPP established a consensus defining probiotics as “live microorganisms that, when administered in adequate amounts, confer a health benefit on the host” (Hill et al., 2014). To exert their beneficial effects, probiotics must be safe for human consumption and resilient enough to withstand GI conditions, ensuring they maintain functionality during digestion. This resilience allows probiotics to support host health by stabilizing the intestinal microbiota and mitigating inflammation (Ojha et al., 2023).

Recent research has focused on the role of probiotics in modulating CNS signaling pathways (Ansari et al., 2023; Kumar et al., 2024). A leading hypothesis suggests that the VN acts as a primary communication conduit between the enteric nervous system (ENS) and the brain, mediated by gut microbiota. This interaction relies on the modulation of neuronal activity via neurotransmitters (Faraji et al., 2024). Specific strains of *Lactobacillus*, *Bacteroides*, and *Bifidobacterium* have been shown to enhance the synthesis of neurotransmitters such as acetylcholine and gamma-aminobutyric acid (GABA), while increasing plasma tryptophan levels, a precursor for serotonin (5-HT) synthesis (Figure 1).

Certain probiotic strains, including *Lactobacillus rhamnosus*, *Bifidobacterium bifidum*, and *Saccharomyces boulardii*, have shown promise in AD models (Un-Nisa et al., 2022). These strains have demonstrated effects such as reducing neuroinflammation and systemic inflammation, strengthening the GI and blood–brain barrier (BBB), and producing metabolites that promote brain health (Figure 1).

#### 1.2.3 Postbiotics

In 2021, the ISAPP defined postbiotics as “preparations of inanimate microorganisms and/or their components that confer a health benefit on the host” (Salminen et al., 2021). Postbiotics contain bioactive compounds such as SCFAs, lactic acid, teichoic acid, antioxidant enzymes, and bacterial cell wall fragments, which exert beneficial effects even in the absence of live bacteria. The potential mechanisms for achieving health benefits through postbiotics resemble those of probiotics (Bashir et al., 2024; Hijová, 2024; Wegh et al., 2019).

A major advantage of postbiotics lies in their stability and extended shelf life, which lowers manufacturing and storage costs while eliminating risks associated with live probiotics, such as virulence factors and antibiotic resistance (Rafique et al., 2023). These characteristics make postbiotics a versatile and promising therapeutic option, particularly for addressing multifactorial conditions like AD.

**Figure 3:**
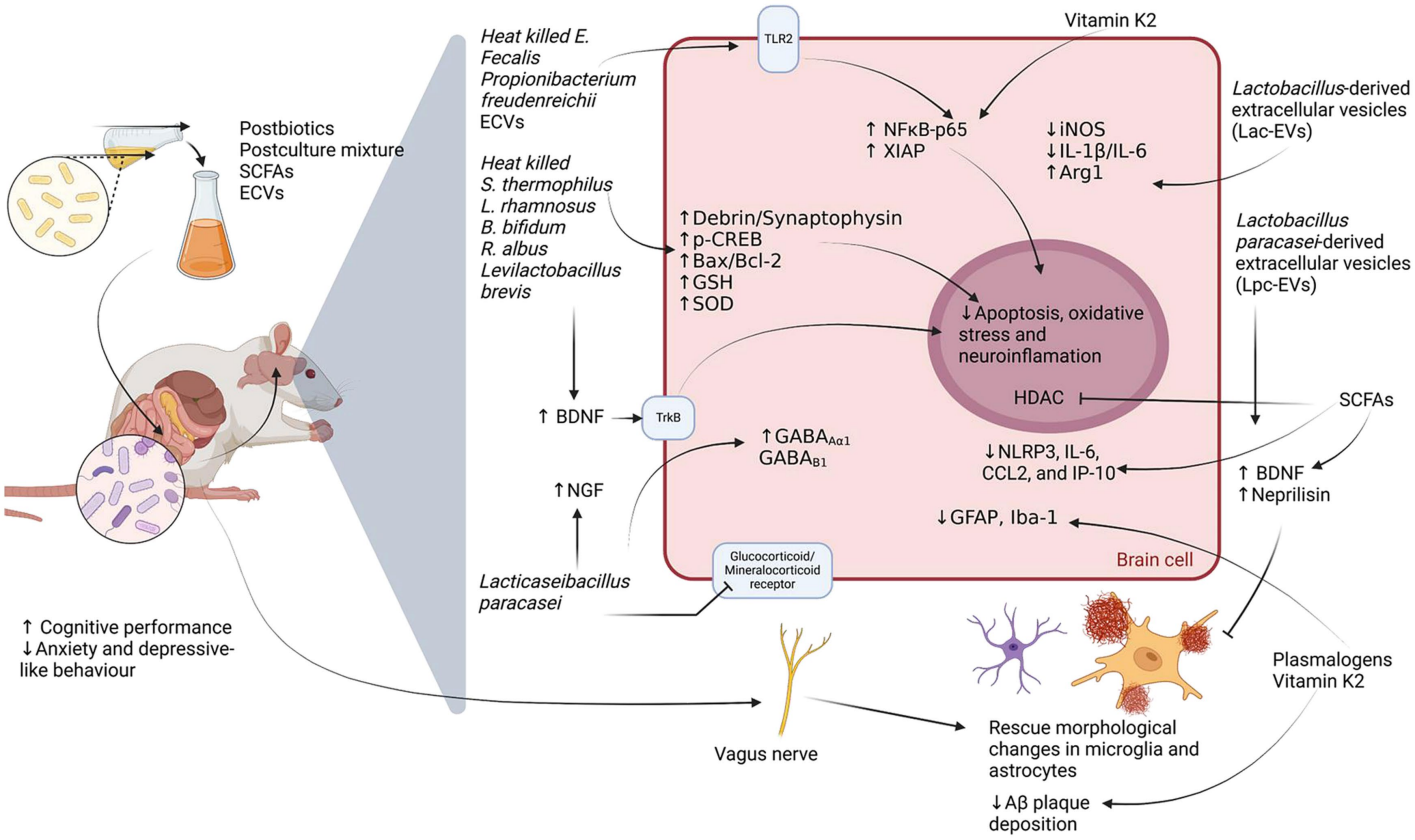
Molecular mechanisms underlying the neuroprotective effects of postbiotics. This figure illustrates the proposed neurobiological actions of postbiotics—such as short-chain fatty acids (SCFAs), postculture supernatants, and bacterial extracellular vesicles (ECVs)—within the microbiota–gut–brain axis in rodent models. Postbiotics have been associated with enhanced cognitive function and reduced anxiety- and depressive-like behaviors. In the central nervous system, they promote neurotrophic support (↑BDNF, ↑NGF), augment antioxidant defenses (↑GSH, ↑SOD), and modulate neurotransmission (↑GABA_Aα1, ↑GABA_Bβ1). They also influence signaling pathways involved in neuroplasticity and inflammation (TrkB, TLR2), potentially attenuating oxidative stress, apoptosis, and neuroinflammatory responses. Moreover, postbiotic interventions are linked to a decrease in pro-inflammatory mediators (↓IL-1β, ↓IL-6, ↓NLRP3, ↓CCL2, ↓IP-10), reduced glial reactivity (↓GFAP, ↓Iba-1), and diminished amyloid-β (Aβ) plaque accumulation, possibly through histone deacetylase (HDAC) modulation and the actions of Vitamin K2, plasmalogens, and bacterial-derived ECVs.

By leveraging mechanisms similar to those of probiotics, postbiotics provide additional advantages, including precise dosing and reduced unpredictability in physiological responses (Rafique et al., 2023). Their pleiotropic effects position them as a valuable tool for mitigating neuroinflammation and supporting cognitive health (Figure 3).

The interaction between the gut microbiota and the brain, mediated by the MGBA, could influence the development of AD by promoting inflammation and oxidative stress. These factors may exacerbate the classic neuropathological features of AD, such as beta-amyloid plaques and tau neurofibrillary tangles.

## 2 Currently cellular and molecular dysfunctions in Alzheimer’s disease

The brains of patients with AD exhibit distinctive neuropathological features, including senile plaques—extracellular aggregates of beta-amyloid protein (A*β*)—and neurofibrillary tangles (NFTs), which are intracellular aggregates of abnormally hyperphosphorylated tau protein. Additionally, a pronounced reduction in cholinergic synapses, particularly within the nucleus basalis, has been extensively documented (DeTure and Dickson, 2019; Maccioni et al., 2001; Mesulam, 2013; Mieling et al., 2023; Morató et al., 2022; Selkoe, 1991; Shankar et al., 2007; Vogels et al., 1990; Whitehouse et al., 1981). Emerging evidence highlights oxidative stress and inflammation as critical contributors to neurodegeneration and neuronal death in AD.

### 2.1 Classic histopathological markers

#### 2.1.1 Senile plaques

The two hallmark neuropathological markers of AD are senile plaques and NFTs. The “amyloid hypothesis” posits that A*β* is the primary driver of AD pathogenesis, with the misfolding of Aβ protein leading to the formation of senile plaques (Cline et al., 2018; Gremer et al., 2017; Hardy and Higgins, 1992; Selkoe and Hardy, 2016). Aβ is derived from the proteolytic cleavage of amyloid precursor protein (APP), a type I transmembrane glycoprotein with a long extracellular glycosylated N-terminal domain and a short cytoplasmic C-terminal domain. The APP gene is located on chromosome 21 and is expressed in glial cells, neurons, and endothelial tissues. Alternative splicing of APP pre-mRNA produces three isoforms: APP770, APP751, and APP695 (Belyaev et al., 2010; Hardy, 1997; Kitaguchi et al., 1988), with the latter being predominantly expressed in the brain and most studied in AD research (Guerreiro et al., 2012; Klein et al., 2016).

APP processing occurs via two pathways: the amyloidogenic and non-amyloidogenic pathways, involving *α*-secretase, *β*-secretase, and *γ*-secretase. In the non-amyloidogenic pathway, α-secretase cleaves APP within the Aβ domain, between residues Lys16 and Leu17, preventing Aβ formation (Esch et al., 1990; Nhan et al., 2015; Sisodia et al., 1990). This cleavage generates a soluble APP ectodomain (sAPPα) and an 83-amino acid C-terminal fragment (C83α). Conversely, the amyloidogenic pathway results in Aβ production through sequential cleavage by β-secretase and γ-secretase (Shoji et al., 1992; Zhang and Song, 2013). β-secretase cleaves APP between Met and Asp1, yielding a soluble ectodomain (sAPPβ) and a 99-amino acid C-terminal fragment (C99). γ-secretase subsequently cleaves C99, producing Aβ peptides of 40 or 42 (Aβ_1-40_; Aβ_1-42_) amino acids and an APP intracellular domain (AICD), which translocates to the nucleus to regulate gene expression, including apoptotic genes.

Aβ deposits are primarily located in the hippocampus, neocortex, and cerebral vasculature (Bloom, 2014; Jellinger, 2002; Viswanathan and Greenberg, 2011). Aβ monomers aggregate into oligomers, which subsequently form insoluble fibrils that assemble into amyloid plaques—a defining feature of AD. While the structure of amyloid oligomers is less well characterized, they are soluble and diffusible, with studies using circular dichroism and infrared spectroscopy indicating that Aβ oligomers adopt β-sheet structures with a stable core (Cerf et al., 2009; Gu et al., 2014; Kayed et al., 2003; Kheterpal et al., 2003). Soluble Aβ interacts with various extracellular molecules, including receptors, metals, and cell membranes, triggering neurotoxic effects. Notably, Aβ oligomers have been implicated in mitochondrial dysfunction, oxidative stress, calcium dysregulation, and neuroinflammation (Bode et al., 2017; Canevari et al., 2004; Huang et al., 2020). These oligomers bind to receptors such as p75 neurotrophin receptors (P75^NTR^), metabotropic glutamate receptors (mGluR), α7 nicotinic acetylcholine receptors (α7nAChR), N-methyl-D-aspartate receptors (NMDA), β-adrenergic receptors (β-AR), and insulin receptors, among others (Cecon et al., 2019; Costantini et al., 2005; Coulson, 2006; De Felice et al., 2007; Liu et al., 2001; Sakono and Zako, 2010; Townsend et al., 2007; Wang et al., 2010; Zhao et al., 2008). These interactions activate neurotoxic signaling pathways, exacerbating oxidative stress and neuroinflammation.

Although the amyloid cascade has long dominated AD research and therapeutic development, mounting evidence challenges the centrality of Aβ as the initiating event in the disease process. Numerous clinical trials aimed at reducing Aβ burden, either by inhibiting its production or promoting its clearance, have failed to yield meaningful cognitive benefits (Doody et al., 2014; Panza et al., 2019). These disappointing outcomes have prompted a re-evaluation of the amyloid hypothesis, suggesting that Aβ accumulation might be a downstream effect of other pathological processes rather than the primary driver of neurodegeneration (Drachman, 2014; Zheng and Wang, 2025). Indeed, emerging models of AD pathogenesis emphasize the contribution of chronic neuroinflammation, mitochondrial dysfunction, oxidative stress, and impaired glucose metabolism to disease progression (Jurcău et al., 2022; Song et al., 2021).

Particularly compelling is the concept of AD as a “type 3 diabetes,” based on the strong association between insulin resistance and AD pathology (de la Monte and Wands, 2008; Peng et al., 2024). This metabolic framework proposes that impaired insulin signaling in the brain contributes to synaptic failure and neurodegeneration, offering a mechanistic link between peripheral metabolic disorders and cognitive decline (Ryu et al., 2019). As such, there is a growing consensus that AD is a multifactorial disorder, necessitating integrated therapeutic approaches that extend beyond Aβ-targeted strategies.

#### 2.1.2 Neurofibrillary tangles

The second hallmark marker, NFTs, is formed by the aggregation of tau protein. Tau is a microtubule-associated protein (MAP) that stabilizes microtubules and plays a critical role in axonal growth and neuronal development (Barbier et al., 2019; Mietelska-Porowska et al., 2014). The tau gene, located on chromosome 17, produces six isoforms in the human brain through alternative splicing. Tau consists of three main domains: an acidic N-terminal domain, a proline-rich central region, and a basic C-terminal domain (Andreadis et al., 1992; Couchie et al., 1992; Goedert et al., 1989; Mietelska-Porowska et al., 2014). The C-terminal domain binds to microtubules to stabilize their assembly, while the proline-rich region is a target for protein kinases. The N-terminal domain interacts with cellular structures such as mitochondria, cytoskeletal elements, or the plasma membrane (Al-Bassam et al., 2002; Brandt et al., 1995; Jung et al., 1993).

Under normal conditions, the phosphorylation of tau is tightly regulated by the balance between tau kinases and phosphatases. In AD, dysregulation occurs, with upregulated kinases and downregulated phosphatases leading to tau hyperphosphorylation (Benítez et al., 2021; Billingsley and Kincaid, 1997). Hyperphosphorylated tau detaches from microtubules, destabilizing them and promoting the formation of insoluble paired helical filaments (PHFs), which aggregate into NFTs. This process contributes to neuronal degeneration and synaptic dysfunction (Dolan and Johnson, 2010; Liu et al., 2005). Key kinases involved in tau phosphorylation include Glycogen Synthase Kinase 3 (GSK3) and Cyclin-Dependent Kinase 5 (Cdk5), among others (Hernandez et al., 2013; López-Tobón et al., 2011; Maldonado et al., 2011; Noble et al., 2003; Plattner et al., 2006). NFTs form within neuronal cell bodies during AD progression and persist after neuronal death, where they may be phagocytosed by astrocytes and microglia.

### 2.2 Oxidative stress

Oxidative stress arises from an imbalance between the production of reactive oxygen species (ROS) and reactive nitrogen species (RNS) relative to the antioxidant defenses of the organism. This phenomenon is a hallmark of various neurodegenerative diseases, with AD being particularly notable (Li et al., 2013; Patten et al., 2010). The accumulation of ROS and RNS triggers signaling pathways that lead to cell death through apoptosis, necrosis, or autophagy.

In AD, Aβ aggregates have been shown to interact directly with lipid and cholesterol components of cell membranes. This interaction disrupts membrane integrity and permeability, forming channels that perturb intracellular calcium (Ca^2+^) homeostasis. These disruptions inhibit long-term potentiation (LTP), induce oxidative and nitrosative stress, and ultimately lead to neuronal death (Bode et al., 2019; De Felice et al., 2007; Mattson and Chan, 2003; Shankar et al., 2007; Townsend et al., 2007; Yasumoto et al., 2019). Additionally, Aβ binds to the NR2B subunit of the NMDA receptor, further deregulating intracellular Ca^2+^ levels, causing synaptic dysfunction and neuronal degeneration (Alberdi et al., 2010; Rönicke et al., 2011; Shankar et al., 2007). These processes collectively exacerbate oxidative and nitrosative stress, driving neuronal degeneration.

The primary ROS and RNS implicated in oxidative stress include singlet oxygen (O_2_), superoxide anion (O_2_^•-^), hydroxyl anion (HO^•^), hydrogen peroxide (H_2_O_2_), nitric oxide (NO^−^), and peroxynitrite anion (ONOO^−^). The brain is particularly susceptible to oxidative stress due to its high metabolic rate and substantial O_2_ consumption. Neurons, unlike other cell types, depend heavily on oxygen for mitochondrial ATP production, but mitochondria are also significant sources of ROS and RNS (Lambert and Brand, 2009). Furthermore, neuronal membranes, rich in polyunsaturated fatty acids and low in antioxidant defenses, are highly prone to lipid peroxidation (Floyd and Hensley, 2002; Mattson et al., 2002).

Elevated levels of Aβ_1-40_ and Aβ_1-42_ peptides correlate with increased oxidative damage to proteins, lipids, and nucleic acids in the hippocampus and cortex of AD patients (Butterfield and Lauderback, 2002). Biomarkers of oxidative stress, such as oxidized proteins, lipids, DNA, and RNA, have been extensively studied in AD (Butterfield et al., 2007). The most critical free radicals involved in oxidative stress are O_2_^•-^, HO^•^, and NO^−^. Neurotoxic mechanisms of oxidative/nitrosative stress mediated by Aβ involve the activation of nitric oxide synthases (NOS), which increase NO^−^ production (Halliwell, 2007). The NOS family comprises three isoforms: neuronal NOS (nNOS), endothelial NOS (eNOS), and inducible NOS (iNOS). Notably, nNOS and eNOS are Ca^2+^-dependent, whereas iNOS functions independently of Ca^2+^. NO^−^ plays crucial roles in central nervous system functions, including learning and memory (Zhou and Zhu, 2009). However, excessive NO^−^ reacts with O_2_^•-^ to form ONOO^−^, inducing nitrosative stress through protein modifications.

ONOO^−^ also promotes lipid peroxidation, damaging cell membranes and generating markers such as malondialdehyde (MDA) and 4-hydroxynonenal (4-HNE) (Di Domenico et al., 2017; Mark et al., 1997; Niedzielska et al., 2016). Lipid peroxidation propagates a chain reaction that alters dendritic and axonal morphology, including myelin sheath damage. Oxidative stress similarly affects DNA, with 8-oxo-2′-deoxyguanosine (8-OHdG) serving as a marker of oxidative DNA damage. Such damage disrupts the cell cycle, protein synthesis, and promotes mutagenesis (Lovell et al., 1999; Nunomura et al., 1999).

The long-term effects of oxidative stress significantly impair cell survival in the CNS. Structures such as the hippocampus, amygdala, and prefrontal cortex are particularly vulnerable. The hippocampus, crucial for spatial learning and memory, is profoundly affected in AD patients, with CA1 neurons and the dentate gyrus exhibiting heightened susceptibility to oxidative damage (Cruz-Sánchez et al., 2010). Additionally, oxidative stress is closely linked to neuroinflammation, as ROS and RNS activate inflammatory pathways in astrocytes, triggering the release of inflammatory mediators like interleukins. This cascade contributes to reactive astrogliosis and microgliosis, further exacerbating neuronal damage (Sudo et al., 2015).

### 2.3 Neuroinflammation

The inflammatory response is intricately modulated by oxidative stress. Under conditions of redox balance, this response functions as a self-limiting defense mechanism that facilitates tissue repair. However, when redox equilibrium is disrupted, the signaling pathways governing the immune system become altered, favoring proinflammatory responses—a hallmark of neurodegenerative diseases (Jayaraj et al., 2017). In the CNS, glial cells, including microglia, oligodendrocytes, and astrocytes, as well as non-glial resident myeloid cells (macrophages and dendritic cells) and peripheral leukocytes, play pivotal roles in mediating the inflammatory response.

Microglia, the resident macrophages of the CNS, constitute approximately 10% of total glial cells in the human brain and are particularly immunologically active in the hippocampal region (Ochocka and Kaminska, 2021; Smith et al., 2012). These cells exhibit a ramified morphology in a healthy brain, indicative of a resting but active state involved in environmental surveillance. Upon activation during pathological conditions, microglia undergo morphological transformations, adopting an amoeboid shape characterized by a larger soma and reduced, retracted processes (Kettenmann et al., 2011; Ransohoff and Perry, 2009; Stence et al., 2001). Activation can be identified by specific surface markers, including Iba-1, IB4, CD11b, F4/80, CD14, CD45, CD80, CD115, and CD68 (Fadini et al., 2013; Jenkins et al., 2013; Jeong et al., 2013; Lin et al., 2005; Rice et al., 2017; Yun et al., 2018; Zanoni et al., 2011). Reactive microglia release cytotoxic factors such as ROS and RNS, which can induce inflammation and transform astrocytes into neurotoxic phenotypes (Liddelow et al., 2017).

Astrocytes, the most abundant glial cells in the brain, are essential for maintaining cerebral homeostasis. They exhibit complex morphologies, with extended processes that interact with neighboring cells (Bélanger and Magistretti, 2009), contacting between 250,000 and 2 million synapses in the human brain (Oberheim et al., 2006). Key astrocytic functions include regulating blood flow, maintaining ionic (e.g., Ca^2+^) and neurotransmitter homeostasis, and serving as the brain’s primary glycogen reserve. These roles are critical for neuronal survival and functionality; thus, astrocytic dysregulation can precipitate neurodegeneration.

Astrocytes exist in either a resting or reactive state. Resting astrocytes vary in morphology depending on their location: fibrous astrocytes, found in white matter, possess long, slender processes, while protoplasmic astrocytes in gray matter have highly branched processes that interface with synapses and blood vessels. Reactive astrocytes, characterized by astrogliosis, exhibit hypertrophic morphology and upregulation of glial fibrillary acidic protein (GFAP). These cells secrete proinflammatory cytokines, such as tumor necrosis factor (TNF) and interleukin-1β (IL-1β) (Giovannoni and Quintana, 2020), as well as ROS. Myoinositol, another astrocytic activation marker, increases during normal aging and in neuroinflammatory conditions (Cabezas et al., 2019; Liddelow et al., 2017; Sofroniew, 2005; Sofroniew, 2009). GFAP and myoinositol serve as reliable indicators of astrocyte activation.

In AD, both microglia and astrocytes contribute significantly to neuroinflammation, which exacerbates neuronal dysfunction and cell death. The bidirectional interactions between these glial cells and their environment underscore the complexity of neuroinflammatory processes and their pivotal role in neurodegenerative diseases.

#### 2.3.1 Neuroinflammation in the microbiota-gut-brain axis

Intestinal dysbiosis is strongly associated with increased intestinal barrier permeability, pathological immunological alterations, and persistent systemic inflammation (Quesada-Vázquez et al., 2022; Zhao M. et al., 2023). Notably, disruption of the intestinal barrier facilitates the translocation of bacterial products, such as lipopolysaccharide (LPS), into the systemic circulation (Ghosh et al., 2020). LPS is recognized by Toll-like receptors (TLRs) on immune cells, triggering an inflammatory cascade characterized by the synthesis and release of proinflammatory cytokines (Ciesielska et al., 2021).

Moreover, dysbiosis results in a decline in beneficial microbiota populations, leading to a reduction in the production of anti-inflammatory metabolites, such as SCFAs (Kandpal et al., 2022). These metabolites, classified as postbiotics, play a pivotal role in modulating the immune system and mitigating chronic inflammation. A deficiency in SCFAs exacerbates immune dysregulation and sustains the inflammatory state (Ney et al., 2023; Zhou et al., 2024).

At the molecular level, several inflammatory pathways are activated because of dysbiosis. One of the most extensively studied is the activation of Toll-like receptor 4 (TLR4) by LPS, which subsequently activates the nuclear factor kappa-light-chain-enhancer of activated B cells (NF-κB) signaling pathway (Ma N. et al., 2023; Zhang S. et al., 2022). This activation results in the production of proinflammatory cytokines, including interleukin-6 (IL-6), tumor necrosis factor-alpha (TNF-*α*), and IL-1β (Vega-Magaña et al., 2020).

Another critical pathway is the Janus kinase/signal transducer and activator of transcription (JAK–STAT) signaling pathway, which is activated in response to cytokines such as IL-6, thereby amplifying inflammatory responses (Li Y. et al., 2024; Uciechowski and Dempke, 2020). Additionally, the nucleotide-binding oligomerization domain-like receptor family pyrin domain-containing 3 (NLRP3) inflammasome plays an essential role in the activation of caspase-1, which promotes the maturation of cytokines IL-1β and interleukin-18 (IL-18) (Pan et al., 2022). Although these pathways are crucial for mounting an immune response against infections, their dysregulated activation contributes to the pathogenesis of chronic inflammatory diseases (Liu et al., 2017; Ozaki et al., 2015).

These interconnected mechanisms underscore the intricate relationship between the intestinal microbiota, immune regulation, and the MGBA. Maintaining microbial homeostasis is thus vital for preventing neuroinflammatory and systemic inflammatory processes, which are linked AD (Giau et al., 2018).

In experimental models, it has been shown that the reduction of microbiome-derived serine/glycine (S/G) lipids decreases the expression of key inhibitors of the inflammatory TLR and NF-κB pathways, thereby increasing proinflammatory responses of systemic monocytes. This imbalance could be related to the dysfunction of other innate immune cells, such as microglia, and contribute to neuroinflammatory pathologies observed in AD (Mihori et al., 2024). Moreover, in models of intestinal microbiota transplantation from patients with AD in APP/PS1 transgenic mice, an increase in the activation of the NLRP3 inflammasome, the release of inflammatory factors and cognitive impairment, accompanied by microglial activation in the hippocampus, has been observed (Shen et al., 2020).

Gut dysbiosis may initiate chronic inflammation through the activation of TLR, NLRP, and JAK/STAT pathways, forging a critical connection to the pathological activation of glial cells, including astrocytes and microglia, in AD. Elucidating the dual roles of glial cells—both protective and harmful—is essential for understanding how neuroinflammatory processes drive disease progression and for uncovering potential therapeutic targets.

## 3 The role of glial cells in Alzheimer’s disease

The activation of inflammatory processes, both systemic and central—including the activation of glial cells—has been extensively studied and is strongly implicated in neurodegeneration (Uddin and Lim, 2022; Zhang W. et al., 2023). Nevertheless, glial cells also play essential roles in the physiological development and maintenance of the CNS. These include synapse formation, maintenance, and function (Allen, 2013); the formation, maintenance, and pruning of myelin sheaths (Hughes, 2021); sustaining energy metabolism (Zhou, 2018); and the establishment, maintenance, and reconstitution of synaptic connections (Auld and Robitaille, 2003).

The main types of glial cells are astrocytes, microglia, oligodendrocytes, and the recently recognized neuron–glia antigen-2 (NG2) glial cells, which have garnered increasing attention in scientific literature (Jin et al., 2018; Peters, 2004). In response to harmful stimuli or pathological conditions within the CNS, microglia are the first to become activated, followed by astrocytes. Astrocytes, in particular, are indispensable for neuronal metabolism, including the regulation of ion balance and neurotransmitter clearance, emphasizing their critical roles in maintaining CNS homeostasis and responding to pathological challenges (Lee et al., 2022; Yang and Zhou, 2019).

Emerging evidence highlights the dual roles of astrocytes and microglia in the CNS (Vainchtein and Molofsky, 2020). These cells exhibit diverse reactive phenotypes influenced by disease stage, type, and regional location within the CNS (Bachiller et al., 2018; Kwon and Koh, 2020). Traditionally, they are classified into neurotoxic (A1/M1) and neuroprotective (A2/M2) phenotypes. However, this binary classification oversimplifies their functional diversity, as glial phenotypes exist along a spectrum (Endo, 2024).

The A2 phenotype of astrocytes and the M2 phenotype of microglia are associated with trophic and protective functions. A2 astrocytes promote neuronal survival and repair by releasing neurotrophic factors and suppressing inflammation. Similarly, M2 microglia contribute to tissue repair, debris clearance, and the secretion of anti-inflammatory cytokines (Kwon and Koh, 2020; Vainchtein and Molofsky, 2020). These phenotypes play crucial roles in mitigating neurodegeneration and fostering recovery, positioning them as potential therapeutic targets for modulating neuroinflammation in AD.

### 3.1 Glial cells as neuroprotective factors in Alzheimer’s disease

Glial cells, particularly microglia, play a crucial role in neuroprotection in AD by mediating the clearance of Aβ plaques through phagocytosis. This process prevents the cytotoxic accumulation of Aβ, a hallmark of AD-associated neurodegeneration (Lee and Landreth, 2010; Wyss-Coray et al., 2003; Yoon and Jo, 2012). Microglia recognize and engulf Aβ deposits via receptors such as TLRs, CX3C chemokine receptor 1 (CX3CR1), and CD36, which facilitate the detection of pathogen-associated molecular patterns (Malm et al., 2015).

A recently identified subset of disease-associated microglia (DAM) has been characterized through single-cell transcriptional analysis. These microglia cluster around Aβ plaques and express genes related to phagocytosis and lipid metabolism, thereby limiting Aβ spread and mitigating its neurotoxic effects. However, chronic exposure to Aβ leads to microglial dysfunction, characterized by a decline in phagocytic capacity and an increase in pro-inflammatory cytokine production, both of which exacerbate neurodegeneration (Wang et al., 2015).

The efficiency of microglia-mediated Aβ clearance declines with aging and chronic inflammation, partly due to the reduced expression of triggering receptor expressed on myeloid cells 2 (TREM2), a key regulator of phagocytosis and inflammation. Notably, genetic variants of TREM2 are associated with an increased risk of AD (Thomas et al., 2022). Recent findings using single-nucleus RNA sequencing have identified both TREM2-dependent and independent microglial responses, underscoring the complexity of microglial activation beyond a simplistic pro- or anti-inflammatory paradigm (Zhou et al., 2020). Additionally, peripheral and perivascular macrophages may contribute to Aβ clearance; however, their effectiveness depends on BBB permeability, which regulates their access to the brain parenchyma (Wen et al., 2024).

### 3.2 Microglia and astrocytes as neurotoxic factors in Alzheimer’s disease

While microglia exhibit neuroprotective functions, they can also contribute to neurotoxicity in AD. The DAM phenotype, initially characterized by its role in Aβ phagocytosis has been linked to genes associated with lipid metabolism, proliferation, and immune activation (Keren-Shaul et al., 2017). Although the existence of a similar microglial phenotype in the human brain remains unclear, several components of the signaling pathways involved in the transition between pro-inflammatory M1 and anti-inflammatory M2 states have been implicated in AD pathophysiology (Gerrits et al., 2021; Guo et al., 2022; Valiukas et al., 2025).

Aβ interacts with pattern recognition receptors (PRRs) such as TREM2, TLR4, and CD14, triggering microglial activation and initiating neurotoxic processes (Colonna and Butovsky, 2017; Heneka et al., 2015). TLR4 activation by Aβ induces downstream signaling cascades, including NF-κB and mitogen-activated protein kinases (MAPKs), leading to the transcription of pro-inflammatory genes and sustaining chronic neuroinflammation (Guo et al., 2024). Additionally, DAM activation amplifies microglial reactivity, reinforcing inflammatory cycles in AD (Wei and Li, 2022).

As pro-inflammatory cytokines and chemokines accumulate, microglial dysfunction results in impaired Aβ clearance, increased Ca^2+^ influx, complement activation, and sustained NF-κB signaling (Muzio et al., 2021; Qie et al., 2020; Wang et al., 2018). These processes not only exacerbate neuronal damage but also compromise the integrity of the CNS microenvironment, including the BBB (Erickson et al., 2012). The BBB, composed of astrocytes, pericytes, microglia, endothelial cells, tight junctions, and a basement membrane, is particularly vulnerable to microglia-driven inflammation. Elevated cytokine levels and microglial phagocytosis of BBB-associated cells have been implicated in its disruption, further aggravating AD pathology (Haruwaka et al., 2019; Mayer and Fischer, 2024).

Astrocytes also play a dual role in AD. Initially, they exert neuroprotective effects, promoting recovery from oxidative stress and inflammation. However, under chronic conditions, their activity shifts, hindering repair mechanisms and exacerbating damage through the release of reactive oxygen species and pro-inflammatory cytokines. The A1 astrocytic phenotype has been specifically associated with the activation of classical complement cascade genes, contributing to synaptic deterioration. Activated microglia induce A1 astrocytes, impairing their physiological functions and promoting neurotoxic activities, ultimately accelerating neuronal and oligodendrocyte loss.

Notably, inhibiting A1 phenotype formation before neuronal injury has been shown to prevent neuronal death, underscoring the critical role of reactive astrocytes in AD pathogenesis. Targeting glial interactions may represent a promising therapeutic strategy to mitigate neuroinflammation and slow neurodegeneration in AD.

Both microglia and astrocytes exhibit profound functional plasticity in response to pathological stimuli, transitioning from protective to neurotoxic states depending on disease stage and microenvironmental cues. While intrinsic CNS signals such as A*β* accumulation are established drivers of glial reactivity, increasing evidence highlights the role of extrinsic factors, particularly those derived from the gut microbiome, in modulating glial activation and cytokine expression.

### 3.3 Microbiota-induced modulation of glial cytokine signaling in Alzheimer’s disease

It suggests that gut microbiota dysbiosis contributes to glial reprogramming and neuroinflammation through systemic immune signaling and microbial metabolites. Disruption of the MGBA facilitates the translocation of microbial products, such as LPS, peptidoglycans, and SCFAs, into circulation, where they interact with peripheral immune cells and can penetrate the central nervous system via a compromised blood–brain barrier (Ashique et al., 2024; Honarpisheh et al., 2022; Solanki et al., 2023).

LPS activates TLR4 expressed on microglia and astrocytes, leading to the activation of NF-κB and MAPK signaling cascades, which in turn induce the transcription and release of pro-inflammatory cytokines including IL-1β, TNF-*α*, and IL-6 (Guijarro-Muñoz et al., 2014). These cytokines perpetuate a state of chronic neuroinflammation, contributing to synaptic dysfunction, oxidative stress, and neuronal apoptosis—hallmarks of Alzheimer’s pathology (Zhang W. et al., 2023). SCFAs, under homeostatic conditions, exert anti-inflammatory effects through GPR41/GPR43 signaling and histone deacetylase inhibition; however, in dysbiotic states, their concentrations and systemic distribution may become imbalanced, failing to restrain glial inflammation (Du et al., 2024; Li et al., 2018).

Additionally, dysbiosis-induced systemic inflammation may increase circulating cytokines that further prime glial cells toward a pro-inflammatory state, lowering the threshold for activation in response to CNS stressors (Solanki et al., 2023). This peripheral-to-central signaling loop underscores the importance of gut microbial composition in shaping the neuroimmune landscape of the AD brain (Anand et al., 2022).

By modulating glial phenotypes and cytokine profiles, the gut microbiota emerges as a key extrinsic regulator of neuroinflammation in AD, offering new insights into therapeutic strategies that target both central and peripheral inflammatory mechanisms.

## 4 Molecular mechanisms of prebiotics, probiotics and postbiotics on glial cells in Alzheimer’s disease

### 4.1 Prebiotics

Prebiotics are non-digestible carbohydrates, primarily polysaccharides and oligosaccharides, that the human body lacks the enzymes to break down (Holscher, 2017). Instead, they reach the intestine intact, where they are fermented by the colonic microbiota. This fermentation process selectively promotes the growth of beneficial bacteria, particularly *Lactobacillus* and *Bifidobacterium*, while inhibiting the proliferation of pathogenic microorganisms (Olveira and González-Molero, 2016; van der Beek et al., 2017; Śliżewska and Chlebicz-Wójcik, 2020) (Figure 2).

Key prebiotics include inulin, FOS, GOS, and xylooligosaccharides (XOS), which differ in their degree of polymerization, ranging from 2 to 10 monomeric units (Broekaert et al., 2011; Marín-Manzano et al., 2013; van de Wiele et al., 2007). Their fermentation enhances the production of SCFAs, such as acetate, which is metabolized in the liver, muscles, kidneys, heart, and brain; butyrate, utilized by commensal bacteria in the colon; and propionate, which is metabolized in the liver and regulates lipid metabolism (Erny et al., 2021; Frampton et al., 2020; Murashige et al., 2020; Song et al., 2019; Tong et al., 2015; Zhao et al., 2020) (Figure 2).

SCFAs play a crucial role in gut health by stimulating mucus production, maintaining optimal pH, accelerating intestinal epithelial healing, and promoting anti-inflammatory responses (den Besten et al., 2013; Fukuda et al., 2011; Vieira et al., 2017). Additionally, they modulate the immune system by reducing pro-inflammatory cytokine production and increasing regulatory T cell populations (Li et al., 2021; Olsson et al., 2021; Sartor, 2014). Given that helper T cells, macrophages, B lymphocytes, and mast cells are major cytokine producers, prebiotics can significantly influence immune regulation. Studies have demonstrated that prebiotic supplementation reduces the levels of pro-inflammatory cytokines such as IL-1*α*, IL-1β, IL-6, IL-12, TNF-α, and IFN-*γ* (Cani et al., 2009; Dehghan et al., 2014; Lecerf et al., 2012).

Neuroinflammation is a key driver of amyloid accumulation and disease progression in AD. However, no effective therapy has been developed to halt its progression. Various factors contribute to AD pathogenesis, including aging, immunosenescence, chronic inflammation, lifestyle habits (diet, exercise, and cognitive engagement), infections, and sleep disorders (Lourida et al., 2013; Ozawa et al., 2014; Sochocka et al., 2019).

Numerous studies highlight the neuroprotective potential of prebiotics. For instance, FOS enhances probiotic survival in the gastrointestinal lumen and exhibits protective effects against Aβ_25-35_ neurotoxicity in PC12 cells. Pretreatment with 10–40 μM Bajijiasu (β-D-fructofuranosyl-(2–2)-β-D-fructofuranosyl) significantly reversed Aβ_25-35_-induced reductions in cell viability and decreased oxidative stress, inflammation, and apoptosis markers (Chen et al., 2013).

Lactulose, a simple prebiotic widely used in commercial formulations, has also shown neuroprotective effects. In a study using an Aβ_25-35_ oligomeric toxicity model in mice, lactulose administration mitigated short-term memory impairment and improved learning ability. The observed neuroprotection was associated with reduced neuroinflammation and enhanced autophagic pathway activation, suggesting lactulose as a potential preventative and/or therapeutic agent for AD (Lee et al., 2021). Similarly, fructooligosaccharides from *Morinda officinalis* (OMO) have demonstrated protective effects against Aβ_1-42_ and Aβ_25-35_-induced cognitive deficits. OMO supplementation improved performance in the Morris water maze, reduced oxidative stress and neuroinflammation, and regulated neurotransmitter release (Chen et al., 2017; Deng et al., 2020).

Metabolic dysfunction has been implicated as a predisposition factor for AD, with some researchers referring to the disease as “type 3 diabetes” due to its links with insulin resistance, neuroinflammation, and cognitive decline (Nguyen et al., 2020; Peng et al., 2024). Early consumption of prebiotics such as inulin may help mitigate this risk. Studies indicate that inulin enhances gut microbiota composition and improves intestinal and brain metabolism in transgenic mice carrying the *APOE4* gene. Additionally, inulin reduces hippocampal expression of inflammatory genes, including *CCL4, CCL10*, and *Fcgr4*, which are associated with Aβ accumulation, vascular damage, and neurodegeneration (Fuller et al., 2014; Tavares et al., 2020; Zhu et al., 2014).

In APP/PS1 transgenic mice, supplementation with FOS, GOS, and a combination of FOS + GOS improved spatial memory and novel object recognition. Among these treatments, GOS was the most effective, followed by FOS + GOS, whereas FOS alone showed no significant effects. Additionally, both the GOS and FOS + GOS groups exhibited reduced expression of Iba-1 and GFAP, markers of microglial and astrocytic activation, respectively. This reduction correlated with lower levels of IL-1β and IL-6, key mediators of neuroinflammation (Zhang S. et al., 2023). Furthermore, these prebiotics modulated the TLR4-Myd88-NF-κB pathway in the colon and cortex. MyD88, a critical adaptor protein, mediates downstream inflammatory signaling, leading to the activation of NF-κB-dependent genes, including those encoding IL-1β, TNF-*α*, IFN-γ, and IL-6 (Cucos et al., 2022) (Figure 2).

XOS have been shown to alleviate cognitive dysfunction and gut microbiota imbalances in APP/PS1 mice undergoing partial hepatectomy. XOS administration reduced peripheral and central levels of IL-1*β* and IL-6, accompanied by decreased Iba-1 expression in the brain. Moreover, XOS treatment preserved blood–brain barrier integrity by upregulating tight junction proteins such as ZO-1 and occludin (Han et al., 2020).

Another promising prebiotic is mannan oligosaccharide (MOS), which has been found to remodel gut microbiota and increase neuroprotective SCFAs. In a study using 5xFAD transgenic mice, MOS supplementation significantly inhibited neuroinflammation and oxidative stress. Researchers observed a reduction in Iba-1 expression in the prefrontal cortex, hippocampus, and amygdala, as well as microglial morphology changes from an amoeboid to a branched form, indicative of decreased activation. Consequently, MOS treatment reduced TNF-α and IL-6 levels (Liu et al., 2021).

The administration of prebiotics and probiotics to maintain or restore gut microbiota composition is emerging as a promising nutraceutical strategy to prevent or mitigate AD symptoms. Recent studies suggest that these compounds can attenuate neuroinflammation and oxidative stress, reducing neurodegeneration in different AD models. Notably, an increase in phagocytic microglia (*CD68+, CX3CR1+*) has been observed in APP/PS1 mice following prebiotic administration, suggesting a neuroprotective effect through enhanced Aβ clearance and immune modulation (Deng et al., 2022; Lana et al., 2024).

### 4.2 Probiotics

The human gut hosts a diverse array of bacterial species and strains, with Firmicutes and Bacteroidetes being the predominant phyla (Buffington et al., 2016). An imbalance in the gut microbiota, known as dysbiosis, has been linked to the development of various diseases, including AD (Vogt et al., 2018). Evidence from germ-free APP/PS1 transgenic mice supports this connection, as these mice exhibit significantly reduced brain Aβ protein levels compared to their conventionally raised counterparts. This finding suggests that the gut microbiota plays a crucial role in neurodegenerative processes (Harach et al., 2017). Moreover, intestinal barrier dysfunction and increased permeability allow microbial metabolites to enter the bloodstream, triggering systemic and central inflammatory pathways. The subsequent disruption of the BBB facilitates the infiltration of proinflammatory cytokines, which activate glial cells and contribute to neuronal damage (Rutsch et al., 2020; Tang et al., 2020). Given their critical role in immune surveillance, microglia are responsible for eliminating pathogens, cellular debris, and Aβ peptides. Studies indicate that probiotic interventions, whether involving single- or multi-strain formulations, can mitigate cognitive deficits in AD animal models by modulating glial cell activity (Lee et al., 2019).

Probiotics are live microorganisms that confer health benefits to their host. They produce antimicrobial compounds, such as bacteriocins, which inhibit pathogenic colonization and enhance the integrity of the intestinal barrier by stimulating mucus production. Additionally, probiotics influence the release of cytokines from epithelial cells, thereby strengthening the immune response (Ohland and Macnaughton, 2010).

In APP/PS1 transgenic mice, a four-week intragastric administration of *Clostridium butyricum* demonstrated significant neuroprotective effects. This probiotic treatment reduced cognitive decline, Aβ deposition, and microglial activation while decreasing TNF-α and IL-1β levels in the brain. Furthermore, *C. butyricum* restored gut microbiota composition and normalized butyrate levels. Notably, butyrate therapy inhibited Aβ-induced NF-κB p65 phosphorylation in BV2 microglia, thereby reducing CD11b and COX-2 expression (Sun et al., 2020). Similarly, in a D-galactose-induced AD model, oral administration of *Lactobacillus plantarum* MTCC 1325 improved cognitive function by restoring acetylcholine (ACh) levels in the hippocampus and cerebral cortex, highlighting the potential of probiotics in modulating gut-brain axis communication (Nimgampalle and Kuna, 2017).

Another study examined the effects of *Lactobacillus pentosus* var. *plantarum* (C29) in a D-galactose-induced aging model. After 5 weeks of administration, C29 reduced cognitive impairment and restored the expression of key neurogenic and anti-inflammatory markers, including brain-derived neurotrophic factor (BDNF), doublecortin (DCX), and cAMP response element-binding (CREB). Notably, C29 significantly suppressed the expression of iNOS, COX-2, p-FOXO3a, p-p65, and the senescence marker p16, suggesting that CREB regulates inflammatory gene expression (Woo et al., 2014). Additionally, C29, when administered in the form of fermented defatted soybeans (FDS DW2009) to 5XFAD transgenic mice, improved cognitive function and modulated microglial activation. This was evidenced by reduced levels of neuroinflammatory markers such as NF-κB, CD11b, and CD11c. Furthermore, C29 favorably altered gut microbiota composition, reinforcing the link between gut health and cognitive function (Lee et al., 2018).

In AD models induced by intracerebroventricular injection of Aβ_25–35_ and Aβ_1–40_, oral administration of *Bifidobacterium breve* A1 (*B. breve* A1) reversed cognitive decline. Gene expression profiling revealed that *B. breve* A1 downregulated inflammation-related genes in the hippocampus and suppressed the transcription of Aβ-induced genes (Kobayashi et al., 2017).

Interestingly, microbial infections, gut dysbiosis, and bacterial byproducts such as LPS have been implicated in AD progression (Fülöp et al., 2020; Kim et al., 2021). A study demonstrated that oral administration of *Lactobacillus mucosae* NK41 and *Bifidobacterium longum* NK46 exerted anti-inflammatory effects on LPS-stimulated macrophages by reducing TNF-α levels and increasing IL-10, an anti-inflammatory cytokine. The combination of these probiotics (NKc) exhibited an even stronger suppression of the TNF-α to IL-10 ratio. In LPS-treated mice, NK41, NK46, and NKc reduced NF-κB + Iba1-positive microglia in the hippocampus, decreased Aβ accumulation, and increased BDNF and IL-10 expression (Ma X. et al., 2023). Additionally, in 5xFAD transgenic mice, these probiotics improved cognitive performance in behavioral tests such as the Y-maze, novel object recognition (NOR), and the Barnes maze. This correlated with lower TNF-α and IL-1β levels, alongside increased IL-10 and BDNF expression in the hippocampus (Ma X. et al., 2023). These findings suggest that NK41, NK46, and NKc can alleviate systemic inflammation and neuroinflammation, thereby mitigating cognitive decline.

Probiotics are widely recognized as a safe and effective natural intervention. However, while their health benefits are well-documented, the U. S. Food and Drug Administration (FDA) mandates further safety studies before approving probiotics for medicinal use.

### 4.3 Postbiotics

Postbiotics and probiotics confer similar benefits through comparable mechanisms, a phenomenon known as the “probiotic paradox” (Adams, 2010). As a result, studies often explore both postbiotics and probiotic secretomes in the context of the MGBA (Figure 3).

One notable study evaluated the effects of heat-killed *Enterococcus faecalis* EF-2001 on inflammatory bowel disease (IBD)-like symptoms and depressive-like behavior in dextran sulfate sodium (DSS)-treated mice. DSS exposure induced significant inflammation and depressive behaviors, characterized by increased TNF-α and IL-6 levels in the rectum and hippocampus, caspase-3 activation, and reduced hippocampal neurogenesis. *E. faecalis* administration reversed these effects by lowering inflammatory cytokine levels and enhancing NFκB-p65 and XIAP expression in the hippocampus. Interestingly, *E. faecalis* increased NFκB-p65 activation in hippocampal astrocytes and microglia, underscoring the crucial role of glial cells in mediating its effects (Takahashi et al., 2019). β-Glucans have been linked to NFκB p65 activation via TLR2 (Aizawa et al., 2018), and *E. faecalis* was found to activate hippocampal TLR2 across multiple cell types. By attenuating DSS-induced neuroinflammation and regulating apoptosis via the NFκB p65/XIAP pathway, *E. faecalis* may offer neuroprotective benefits, particularly through its interaction with hippocampal microglia (Takahashi et al., 2019).

A separate study investigated the effects of different doses of live and inactivated *Streptococcus thermophilus* MN-ZLW-002 in APP/PS1 male mice, demonstrating significant cognitive improvements, including enhanced learning and memory. These benefits were associated with increased hippocampal mRNA levels of superoxide dismutase (SOD) and BDNF. Additionally, *S. thermophilus* administration altered gut microbiota composition, promoting colonic propionic acid-producing bacteria. However, increased systemic inflammation, evidenced by elevated serum inflammatory markers, may have contributed to heightened gliosis in the hippocampus (Zhang et al., 2024). The observed rise in colonic propionic acid-producing bacteria suggests a potential microbiome-gut-brain axis interaction, given that *S. thermophilus* produces SCFAs, which influence BDNF expression and downstream signaling pathways(Church et al., 2023).

TrkB receptors, expressed in neurons, astrocytes, and microglia, are pivotal in BDNF signaling, which is essential for CNS function. Upon BDNF binding, TrkB activation triggers intracellular pathways such as PI3K/Akt, MAPK/ERK, and PLC*γ*, leading to enhanced cell survival, differentiation, and synaptic plasticity (Rosso et al., 2020; Wang et al., 2022; Wang et al., 2024). SCFAs, generated through the anaerobic fermentation of dietary fiber by gut microbiota, have emerged as key mediators of gut–brain communication. Increasing evidence indicates that SCFAs contribute significantly to the maintenance of blood–brain barrier (BBB) integrity, particularly under pathological conditions (Fock and Parnova, 2023; Liu et al., 2015). Additionally, SCFAs can cross the BBB and promote neuroprotective mechanisms, further supporting cognitive function (Church et al., 2023; Zhao T. et al., 2023). By modulating inflammation and enhancing neuroprotection, postbiotics could improve neuronal health and cognitive function.

Reduced expression of tight junction proteins, including claudin-5 and occludin, has been observed in germ-free mice, indicating compromised BBB integrity. This phenotype is reversible upon colonization with defined bacterial consortia containing SCFA producing strains (Braniste et al., 2014). The presence of free fatty acid receptor 3 (FFAR3) in brain endothelial cells further suggests a mechanism through which butyrate may regulate BBB structure and function (Hoyles et al., 2018). SCFAs are primarily absorbed via co-transport with Na^+^ or K^+^ ions (Stumpff, 2018). Their ability to cross the BBB was demonstrated by carotid artery injection of radiolabeled 14C-SCFAs in rats, with detectable levels in brain tissue (Oldendorf, 1973). Among these, butyrate exhibited the highest brain uptake, followed by propionate and acetate. Transport across the BBB is mediated predominantly by monocarboxylate transporters (MCTs), particularly MCT1 and MCT4, members of the SLC16 family (Pellerin et al., 2005; Pierre and Pellerin, 2005; Terasaki et al., 1991). These proton-coupled transporters facilitate SCFA entry from circulation into the CNS (Halestrap and Price, 1999). MCT1, with higher substrate specificity, transports acetate and butyrate and is expressed in cortical and hippocampal astrocytes, endothelial cells, and pericytes (Baud et al., 2003; Gerhart et al., 1998; Hanu et al., 2000; Pellerin et al., 1998; Pierre et al., 2000; Vannucci and Simpson, 2003) (Figure 2). Although passive diffusion of SCFAs in their non-ionized form is possible, it is limited under physiological conditions due to their polarity, highlighting the essential role of transporter-mediated uptake.

Despite extensive research on the intestinal and neural effects of inactivated bacteria, data on their modulation of glial cells remain scarce. However, multiple studies have documented the health-promoting properties of postbiotics. For instance, the effects of heat-killed *Lacticaseibacillus paracasei* N1115 were tested in an antibiotic-induced dysbiosis (Abx) model in neonatal male mice. Over 84 days, *L. paracasei* treatment improved cognitive performance, anxiety, depressive-like behaviors, and locomotor activity. In the Morris Water Maze, N1115-treated mice exhibited reduced escape latency, while the Tail Suspension Test revealed shorter immobility periods, and the Open Field Test demonstrated increased motor activity compared to the Abx group. Moreover, *L. paracasei* administration was associated with normalized serum levels of IL-1, IL-6, and corticosterone, akin to control animals (Zhang Y. et al., 2022).

Previous studies have reported the anxiolytic and antidepressant effects of heat-inactivated bacteria, suggesting that postbiotics regulate stress hormones through modulation of the hypothalamic–pituitary–adrenal (HPA) axis (Kambe et al., 2020; Maehata et al., 2019; Warda et al., 2019). The exact mechanisms underlying these behavioral improvements remain unclear, yet they may involve neurochemical and neurotrophic factor regulation in the hippocampus and prefrontal cortex. *L. paracasei* treatment mitigated glucocorticoid receptor-induced stress, upregulated nerve growth factor (NGF) expression, and downregulated mineralocorticoid receptor (MR) levels, indicating restored neurotrophic support and balanced HPA axis signaling. Additionally, it counteracted dysbiosis-induced alterations in neurochemical signaling, reversing changes in GABAA*α*1, GABAB1, and BDNF expression (Zhang Y. et al., 2022).

One of the most consistent findings in postbiotic research is their ability to restore microbial eubiosis. This has been demonstrated in the bifidogenic properties of heat-killed *Limosilactobacillus vaginalis in vitro* (Giordani et al., 2023). This is particularly relevant in cesarean-section births, where neonates lack exposure to beneficial vaginal bacteria, resulting in reduced gut microbial diversity and increased susceptibility to diseases. Heat-killed *L. vaginalis* could potentially reinstate microbiome integrity by promoting *Bifidobacterium* spp. (Giordani et al., 2023), a key factor in neuroimmune regulation of glial cells (Erny et al., 2015).

Glial activation by the microbiome is mediated partly through the vagus nerve (Martin et al., 2018). Ultrasound-guided percutaneous vagus nerve stimulation has been shown to restore microglial and astrocyte morphology, reduce A*β* plaque deposition in the hippocampus, and modulate the autophagy-lysosomal pathway by upregulating transcription factor EB (TFEB) and lysosomal-associated membrane protein 1 (LAMP1) expression. Furthermore, microbiome integrity plays a crucial role in hippocampal neurogenesis (Ogbonnaya et al., 2015).

Bacterial extracellular vesicles (EVs) are also emerging as promising regulators of neuroinflammation (Pirolli et al., 2021). *Lactobacillus*-derived EVs (Lac-EVs) were found to suppress LPS-induced inflammation in microglia by downregulating iNOS (M1 phenotype marker) and upregulating Arg1 (M2 phenotype marker), shifting microglia toward an anti-inflammatory profile. Lac-EVs also reduced pro-inflammatory cytokine secretion (IL-1β, IL-6) while increasing IL-10 production (Yang et al., 2024).

Similarly, *Lactobacillus paracasei*-derived EVs (Lpc-EVs) demonstrated neuroprotective effects in an AD model (Kwon et al., 2023). Lpc-EVs mitigated Aβ-induced neurotoxicity in HT22 cells and APP/PS1 mice by upregulating neurotrophic factors (BDNF, Nt3, Nt4/5) and the TrkB receptor while modulating MeCP2 and Sirt1 expression. Additionally, Lpc-EVs restored Aβ-degrading proteases (Mmp-2, Mmp-9, Nep) and reduced Aβ accumulation and neuroinflammation, thereby improving cognitive function (Kwon et al., 2023).

SCFA depletion during aging disrupts the BBB and impairs microglial function, contributing to CNS damage (Knox et al., 2022). SCFAs are among the most studied postbiotic compounds, with evidence showing their ability to mitigate hypoxic–ischemic brain injury (HIBI) by reducing astrocyte activation and preserving oligodendrocyte precursor cells (OPCs). SCFAs also downregulate pro-inflammatory markers (NLRP3, IL-6, CCL2, IP-10) and regulate apoptosis-related proteins (Bax, Bcl-2), suggesting a protective role against neuroinflammation (Gao et al., 2022).

Certain strains of lactic acid bacteria produce menaquinones (vitamin K2) as part of their fermentation process (Morishita et al., 1999). These molecules play a crucial role in sphingolipid synthesis, which is essential for maintaining the structural integrity and stability of the myelin sheath, a key component in cell signal transduction. The synthesis of sphingolipids depends on vitamin K-dependent enzymes (Giussani et al., 2021).

*In vitro* studies have demonstrated that vitamin K2 derivatives can mitigate the amyloidogenic pathway by modulating genes involved in the toxic processing of the APP. This modulation results in the downregulation of beta-site APP cleaving enzyme 1 (BACE1) and presenilin 1 (PSEN1) expression, which encode for the active sites of β- and γ-secretases, respectively. In transgenic mouse models, these proteins are significantly upregulated, leading to excessive Aβ production and aggregation (Hampel et al., 2021). Interestingly, menaquinone treatment has been shown to enhance the expression of secretases involved in the non-amyloidogenic pathway, as evidenced by increased A Disintegrin And Metalloprotease 17 (ADAM17) levels compared to control groups (Orticello et al., 2023).

Another *in vitro* study investigated the effects of vitamin K2 on c-Myc-immortalized MG6 microglial cells, revealing a dose-dependent reduction in the mRNA expression of pro-inflammatory cytokines such as IL-1β, TNF-*α*, and IL-6. This anti-inflammatory effect was observed in microglial cultures stimulated with LPS and TNF-α. Typically, these inflammation cascades are mediated by NF-κB, a transcription factor activated in response to toxic pro-inflammatory molecules (Bubici et al., 2006). The study suggested that menaquinones may inhibit NF-κB translocation by promoting p65 phosphorylation, thereby suppressing its transcriptional activity (Saputra et al., 2019). Similarly, bacterial EVs derived from *Propionibacterium freudenreichii* exhibit potential anti-inflammatory properties by modulating the NF-κB pathway. This effect appears to be partially mediated by surface-layer protein B (SlpB) and other cytoplasmic factors that interact with LPS-NF-κB signaling pathways (Rodovalho et al., 2020). However, it remains unclear whether *P. freudenreichii* EVs can cross the BBB to exert direct neuroprotective effects. Nevertheless, previous studies indicate that certain bacterial nanosized EVs can communicate with the brain (Pirolli et al., 2021), suggesting that their role in CNS modulation warrants further investigation.

Plasmalogens (Pls), another class of bacterial metabolic products, have been identified in postbiotic mixtures (Řezanka et al., 2012). Their therapeutic potential in AD is particularly relevant, as Pls levels are notably reduced in this pathology (Wood et al., 2010). Studies suggest that plasmalogen supplementation can mitigate cognitive decline in AD (Fujino et al., 2017; Hossain et al., 2018). For instance, in an LPS-induced neuroinflammation mouse model, 3 months of PLS treatment significantly improved memory performance compared to control animals. This cognitive recovery correlated with reduced GFAP and Iba-1 immunoreactivity, as well as lower A*β* burden in astrocytes and neurons within the prefrontal cortex and hippocampus (Hossain et al., 2018). Similar cognitive improvements were observed in a randomized, double-blind, placebo-controlled clinical trial involving mild AD patients who received plasmalogen supplementation (Fujino et al., 2017).

Other postbiotic metabolites, such as nicotinamide N-oxide (NAMO), equol, and linoleic acid derivatives, have also demonstrated neuroprotective and antioxidant properties. These compounds are known to modulate glial-cytokine interactions, which may contribute to their therapeutic potential in neurodegenerative diseases (Ikeguchi et al., 2018; Johnson et al., 2020; Li et al., 2022; Song et al., 2022). Increasing evidence supports the role of postbiotics in CNS health, and their mechanisms have been extensively reviewed elsewhere (Głowacka et al., 2024).

Emerging research highlights the significant potential of postbiotics in modulating neuroinflammation and enhancing cognitive function in neurodegenerative disorders. These effects are primarily mediated through the regulation of pro- and anti-inflammatory cytokines, neurotrophic support, and antioxidant activity. However, the precise interactions between postbiotics and glial cells remain largely unexplored, underscoring the need for further investigation to elucidate the complex cellular and molecular mechanisms underlying their neuroprotective effects.

## 5 Synergistic interventions: the role of synbiotics in Alzheimer’s disease

The term synbiotic refers to the synergistic combination of probiotics and prebiotics, formulated with the aim of enhancing the viability and functionality of probiotic strains through the provision of their specific substrates (Gibson and Roberfroid, 1995; Swanson et al., 2020). This interaction promotes colonization of the gastrointestinal tract and beneficial metabolic activity, thereby optimizing the impact on the gut microbiota (Yadav et al., 2024). In this context, a well-designed synbiotic formulation may induce superior effects compared to those obtained from individual components, positioning it as a promising multi-target therapeutic strategy for neurodegenerative disorders such as AD (Ngoc et al., 2024).

Although preclinical and clinical studies evaluating synbiotics in AD models remain limited, emerging data suggest significant benefits (Bonfili et al., 2025; Ton et al., 2020). In parallel, animal studies employing fecal microbiota transplantation and microbiome profiling have shown that modulation of gut microbial composition can influence cognition, neuroinflammation, and amyloid pathology in AD models (Kim et al., 2020; Sun et al., 2019). Their administration has been reported to improve cognitive performance, potentially by attenuating gastrointestinal inflammation, reducing oxidative stress, and modulating neuroinflammatory processes in key brain regions such as the hippocampus, which is involved in learning and spatial memory (Li L. et al., 2024; Ton et al., 2020; Tong et al., 2024). In an uncontrolled clinical study, researchers demonstrated that daily supplementation with kefir-fermented milk for 90 days in Alzheimer’s disease patients resulted in marked cognitive improvements, along with reductions in systemic inflammation, oxidative stress markers, and blood cell damage—suggesting kefir may serve as a promising adjuvant therapy in mitigating AD progression (Ton et al., 2020). These findings support the hypothesis that synbiotics may be more effective than probiotics or prebiotics alone, particularly in modulating the gut-brain axis.

However, their use is not without risks. Adverse effects such as sepsis, hypersensitivity reactions, and horizontal gene transfer, potentially contributing to antimicrobial resistance, have been reported (Ngoc et al., 2024). This is particularly concerning in immunocompromised patients, such as those undergoing immunosuppressive therapies, where cases of systemic fungal infections and sepsis associated with *Saccharomyces boulardii* administration have been documented (Dudek-Wicher et al., 2020). Certain probiotic strains have also been shown to harbor antibiotic resistance genes, posing additional clinical risks. Moreover, specific metabolic interactions, such as involvement in the tryptophan pathway, may increase the risk of serotonin syndrome when combined with selective serotonin reuptake inhibitors (SSRIs) (Arora et al., 2020). Despite the limited number of studies, these observations underscore the need for rigorous safety evaluations, particularly in AD patients with multiple comorbidities and concurrent pharmacological treatments.

## 6 Translational limitations of preclinical models of Alzheimer’s disease and their interaction with the microbiota-gut-brain axis

Despite significant advances in AD research using preclinical models, substantial translational gaps persist. Transgenic mouse models recapitulate discrete pathological features—such as A*β* accumulation or tau hyperphosphorylation—but fail to reflect the full chronobiological, genetic, and clinical complexity of human AD. Most strains exhibit accelerated disease progression and lack key human risk factors, including advanced age, sex-specific vulnerability, and metabolic or vascular comorbidities (Granzotto et al., 2024; Mullane and Williams, 2019).

A critical additional challenge lies in the unsystematic evaluation of microbial interventions. Studies vary widely in probiotic or symbiotic strains used, dosing regimens, treatment duration, administration routes, and model conditions, hindering cross-study reproducibility and comparability. Moreover, essential variables—such as baseline microbiota composition, gut permeability, and host immune status—are often inconsistently addressed (Ferreiro et al., 2023; Segers and Lebeer, 2014). The lack of methodological standardization and limited translational validation call for cautious interpretation. Addressing these limitations is vital to design robust, clinically relevant studies that can advance gut-brain axis research in AD.

## 7 Conclusion

Inflammation is a hallmark of numerous neurodegenerative and peripheral diseases. In AD, the progressive decline in cognitive functions, such as learning and memory, is closely linked to increased production of ROS, RNS, and pro-inflammatory mediators. Growing evidence indicates a significant elevation of inflammatory molecules in the brain, cerebrospinal fluid, and peripheral blood of AD patients, including IL-1β, IL-6, IL-10, and TNF-α, among others. Additionally, gut microbiota dysbiosis has emerged as a potential contributor to AD pathogenesis by increasing intestinal permeability, exacerbating local inflammation, and promoting neuroinflammation. Although further research is needed to establish a definitive causal relationship between gut microbiota alterations and AD, current findings suggest that dysbiosis may accelerate disease onset and progression. These insights pave the way for novel therapeutic strategies involving prebiotics, probiotics, and postbiotics. As discussed throughout this review, these compounds exert their effects primarily by modulating inflammation both peripherally and centrally. Future studies should aim to elucidate the precise mechanisms linking gut microbiota to AD, which may lead to more effective therapeutic interventions—an urgent need given the limited treatment options currently available.
